# “It was almost like it’s set up for people to fail” A qualitative analysis of experiences and unmet supportive needs of people with Long COVID

**DOI:** 10.1186/s12889-023-17033-4

**Published:** 2023-10-31

**Authors:** Katherine C. McNabb, Alanna J. Bergman, Rhonda Smith-Wright, Jaime Seltzer, Sarah E. Slone, Tosin Tomiwa, Abeer Alharthi, Patricia M. Davidson, Yvonne Commodore-Mensah, Oluwabunmi Ogungbe

**Affiliations:** 1 Center for Infectious Disease and Nursing Innovation, Johns Hopkins University School of Nursing, 525 N. Wolfe St., Baltimore, MD 21205 USA; 2 Johns Hopkins University, Johns Hopkins School of Nursing, Baltimore, USA; 3 Stanford University, Stanford School of Medicine, Palo Alto, USA; 4 The Myalgic Encephalomyelitis Action Network, Santa Monica, USA; 5 Johns Hopkins University, Johns Hopkins Institute for Clinical and Translational Research, Baltimore, USA; 6 University of Wollongong, Wollongong, Australia; 7 Johns Hopkins University, Bloomberg School of Public Health, Baltimore, USA; 8 Johns Hopkins University, Johns Hopkins School of Medicine, Baltimore, USA

**Keywords:** PASC, Long-COVID, Support, Chronic, Occupation

## Abstract

**Background:**

Almost twenty percent of adults with COVID-19 develop Long COVID, leading to prolonged symptoms and disability. Understanding the supportive needs of people with Long COVID is vital to enacting effective models of care and policies.

**Design/methods:**

This qualitative sub-study explored the experiences of people with Long COVID and their unmet needs. Participants enrolled in a larger study to evaluate the post-acute cardiovascular impacts of COVID-19 were invited to participate in subsequent in-depth interviews. Participants were enrolled purposively until saturation at 24 participants. Data were analyzed using thematic content analysis.

**Results:**

Participants focused on adaptations to life with Long COVID and their unmet needs in different life spheres. Three domains, 1) occupational and financial; 2) healthcare-related; and 3) social and emotional support, emerged as areas affecting quality of life. Although participants were motivated to return to work for financial and personal reasons, Long COVID symptoms often resulted in the inability to perform tasks required by their existing jobs, and unemployment. Those who maintained employment through employer accommodations still needed additional support. Participants encountered diagnostic challenges, challenges in accessing specialty appointments, insurance loopholes, high healthcare costs, and medical skepticism. Existing social networks provided support for completing daily tasks; however, those with Long COVID typically turned to others with similar lived experiences for emotional support. Participants found government support programs inadequate and difficult to access in all three domains.

**Discussion:**

We propose a five-pronged policy approach to support persons with Long COVID. These overarching recommendations are (1) improve public awareness of Long COVID; (2) improve clinical care quality and access; (3) implement additional school and workplace accommodations; (4) strengthen socioeconomic benefits and social services; and (5) improve research on Long COVID.

**Supplementary Information:**

The online version contains supplementary material available at 10.1186/s12889-023-17033-4.

## Introduction

According to the World Health Organization (WHO), over 750 million SARS-CoV-2 infection (COVID-19) cases have been confirmed globally, leading to almost 7 million deaths [1] Over 1 million deaths have occurred in the U.S. alone [2]. Although the United States government and the WHO have recently declared the end of COVID-19 as a public health emergency [3, 4], the ramifications on both the individual and societal levels continue to be significant. While the severity of acute impact of COVID-19 has waned due to vaccines [5], treatments [6], and new variants [7], COVID-19 has precipitated a range of ongoing physical, psychological, and social effects, many of which are not yet fully understood [8, 9].

The median time between onset and resolution of symptoms related to COVID-19 is roughly two weeks [10]. Yet the burden of post-acute sequelae of COVID-19 infection (PASC) is substantial. Nearly one in five US adults previously infected with COVID-19 have developed PASC [11]. While PASC terminology and standardized case definition are still in evolution, the affected community has claimed the term “Long COVID” and the sequelae are generally defined as new, persistent, or recurrent symptoms four weeks post-acute COVID-19 infection [12, 13]. Common symptoms of Long COVID include fatigue, post-exertional malaise (PEM), shortness of breath, cognitive dysfunction, and other symptoms that may be new-onset after acute COVID-19 or persist from the initial illness [14].

Early in the pandemic, many medical providers were unaware that patients might experience chronic symptoms after COVID-19 due to an unfamiliarity with infection-associated chronic illnesses. This knowledge deficit resulted in some medical providers dismissing Long COVID symptoms and delaying diagnosis. This is similar to stigma and dismissal associated with other post viral syndromes [15]. Some patients sought multiple medical consultations in search of diagnosis and treatment [16]. Diagnosis of Long COVID is challenging as it is not broadly clinically recognized, and there is no definitive diagnostic test. Diagnosis therefore requires systematic exclusion of differential diagnoses [17–19]. Ultimately, the initial diagnosis of Long COVID may be based on history and physical exam.

Little is known about the long-term impact of COVID-19 and the variety of PASC experiences, management, and recovery, including psychological and socioeconomic impact [19, 20]. Of the existing studies, several have explored decreased quality of life and found negative impact on ability to maintain employment; however, research is limited [16, 21–23]. People have conveyed a variety of experiences in regard to initial infection, isolation, protracted illness, and improvement of post-acute sequelae [24].

Although legislators, advocates, healthcare workers, and those with Long COVID within the United States recognize the need to adapt current public benefit programs and workplace accommodation policies to better support those with Long COVID, there is little existing research to inform new policy recommendations. This study aimed to explore existing and desired supports that facilitate improvement in Long COVID symptoms and, where possible, reintegration into social and professional spheres to inform practice and policy to better support people experiencing Long COVID.

## Methods

This paper represents a qualitative arm of a larger, multi-method research study underpinned by a pragmatic epistemology. The purpose of the larger study was to characterize the cardiovascular impact of Long COVID on people living with and without pre-existing cardiac disease [25]. Participants were recruited for the parent study via the Johns Hopkins University HOPE registry (Hopkins Opportunities for Patient Engagement registry) which solicits patient engagement through a variety of modalities including texts, social media, the electronic medical record, and MyChart. Individuals enrolled in the parent study were approached about their interest in participating in a qualitative sub-study. Participants were initially enrolled consecutively, and then later, researchers used purposive sampling to maximize variability in gender and COVID-19 related symptoms consistent with the parent study aims. At baseline, 63 individuals were interested in participating in the qualitative study. Recruitment was halted at 26 participants when redundant themes were identified indicating sufficient saturation as determined by the primary investigator who reviewed transcripts on an ongoing basis [26]. Participants in the qualitative study were contacted by a study team member to schedule an interview via Zoom at a mutually convenient time. Participants elected whether to appear on video based on their comfort, but Zoom video recording was disabled to increase confidentiality. All interviewers were PhD students formally trained in qualitative research and interview techniques. The semi-structured interview guides were designed to elicit information on participants’ past medical histories, baseline physical health and wellness routines, and the impact of Long COVID on work, finances, and relationships. Each interview was attended by the participant, an interviewer, and a note-taker – a study team member who collected information on non-verbal cues, emotional reactions, and feelings that are not captured in a written transcript. Interviews were audio recorded and professionally transcribed. Each participant received a $25 gift card to compensate for their time and effort. This study protocol was approved by an Institutional Review Board; written informed consent was obtained from all subjects involved in the study.

For this qualitative sub-study, we adopted a critical realist ontology to explain a reality that exists within a bias-laden cultural value system. A critical lens helped guide inquiry about institutional and structural barriers to health that are often beyond the control of individuals [27, 28]. Three members of the qualitative research team divided the transcripts and read through them repeatedly to develop a deep familiarity with the data. After the initial review phase, the coding team developed an inductive codebook from the data and met weekly to discuss memos, reflections, and potential sources of bias. New codes were added to the codebook iteratively as new concepts and potential themes emerged. Every fifth interview was double coded for consistency and to evaluate coding agreement. We used thematic content analysis to group findings using constant comparison to triangulate the data and enhance confirmability [29].

## Results

Twenty-four participant interviews were included in the final analysis; we excluded two qualitative participants because those individuals did not report their lived experiences with Long COVID. Most (79%) were White adults, and 52% of participants were female. The median age of the interviewed participants was 46.5 years. Most participants reported their employment status as full-time, although this did not capture those who were on short-term disability, sick leave, or modified/hybrid schedules. The demographic makeup of the sample is presented in Table 1.

**Table 1:** Participant demographics

	**n (%)**
**Total**	24 (100)
**Gender**	
Male	11 (45)
Female	13 (54)
**Age, years** ^a^	46.5 (39.7, 55)
**Race**	
White	20 (79)
Black	2 (8)
Asian	2 (8)
**Ethnicity**	
Latin/Hispanic/Spanish descent	3 (12.5)
Non-Latin/Hispanic/Spanish	21 (87.5)
**Educational status**	
Graduate	9 (38)
Bachelors	9 (38)
Some College	5 (21)
High School/ GED	1 (8)
**Employment**	
Full time	16 (66)
Part time	2 (8)
Unemployed or disabled	3 (13)
Retired	3 (13)
**Healthcare worker**	
Yes	5 (21)
No	19 (79)
**Household Income**	
≥ 100,000 USD	7 (29)
70,000–99,000 USD	7 (29)
40,000–69,000 USD	5 (21)
< 39,000 USD	3 (13)
Prefer not to answer	2 (8)
**Covid-19 Hospitalization**	
Yes	5 (21)
No	19 (79)

^a^Median and Interquartile Range Reported

**Fig. 1:**
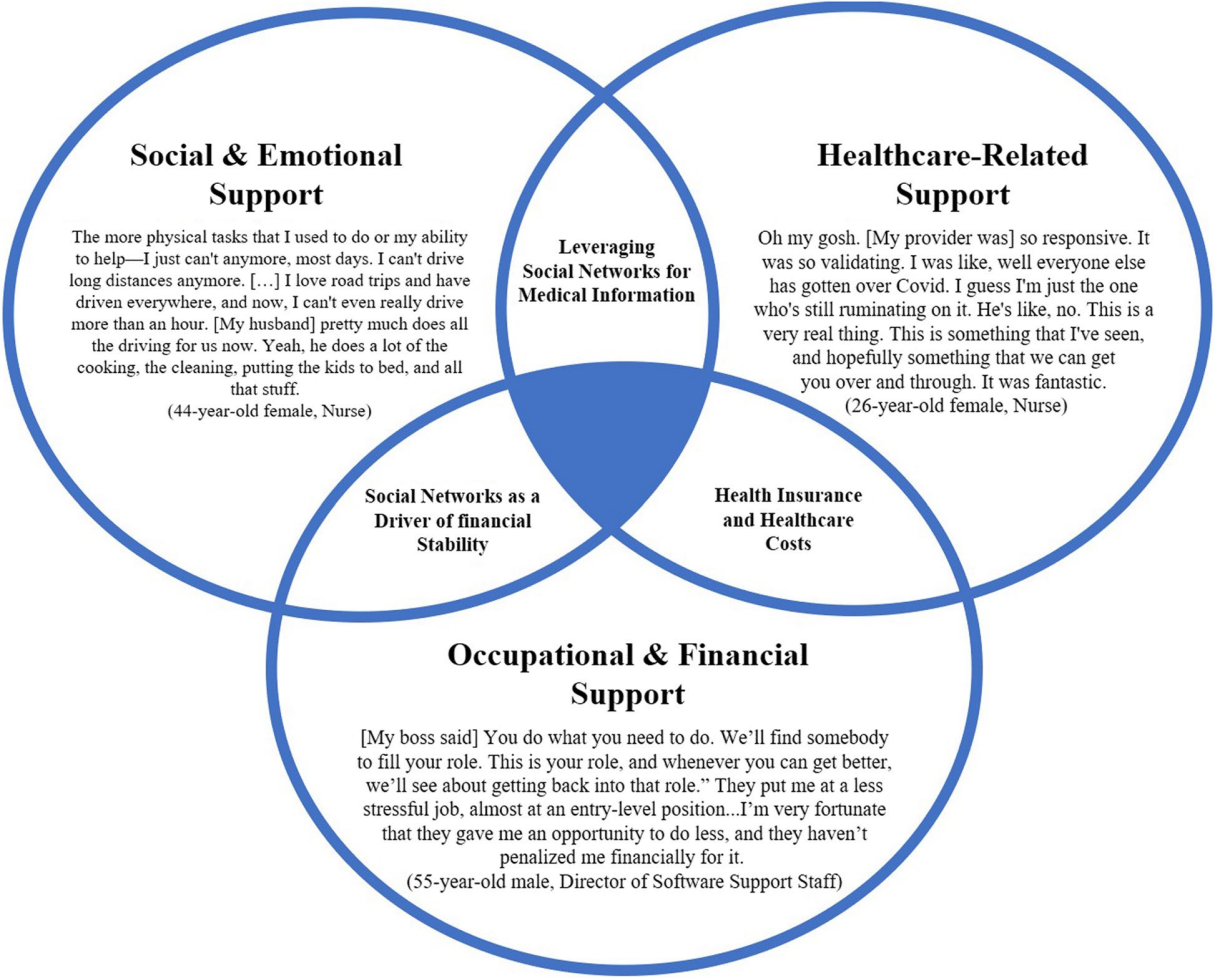
Overlapping Dimensions of Support for People with Long COVID

Consistent with themes that emerged during data analysis, three domains of support were chosen for additional exploration and interpretation: (1) occupational and financial support; (2) social and emotional support; and (3) healthcare support are presented in Fig. 1 and discussed below.

### Occupational and financial support

Participants expressed frustration in some circumstances and gratitude in others regarding their employers’ responses and accommodations to Long COVID symptoms. Some participants indicated that their employers were sympathetic and attempted to increase accessibility but did not understand their needs as chronically ill employees. Participants wanted to continue work that was fulfilling and that helped them feel productive.

*I've never loved a job the way I loved my job. I worked with great people, but I love the patients, and the patients love me, and that was important to me... My identity is nebulous right now because I don't know if I'm ever going to be able to practice, in any way. (44-year-old female, Nurse)*

However, participants who did maintain their prior positions often felt inadequately supported. People with Long COVID lacked the time and physical space to attend to their symptoms and health needs. Short breaks that did not allow for significant rest and recovery, few opportunities to sit at work, and a lack of privacy were some of the barriers to maintaining occupational roles post-COVID.

*It’s a pretty physically demanding job, no matter what you do. It’s a lot of walking, and this Long COVID has basically made working there almost impossible because when I come home from work, forget it—I don’t even bother to eat. I’ve just got to lay down and go to sleep because I’m so tired, and we get breaks, but they aren’t what you would call normal people breaks. We get 15 minutes and 30 minutes here and there, and that’s like 15 seconds to me (63 year-old male, Warehouse Associate)*

Across interviews, people with Long COVID had variable symptom severity, describing a constellation of symptoms that ebbed and flowed. Participants described having good days where they felt able to commute and work in person but also bad days where they needed time and space to rest for prolonged periods. As a result, individuals who were able to work from home acknowledged that remote work was key to maintaining their employment and financial stability. Similarly, people with Long COVID symptoms who were unable to work remotely described difficulty in maintaining employment due to unpredictable symptom presentation.

*I have to do a lot of work in the field, the community, remember: [blinded for confidentiality] city. It was very hard for me just to come into the place... because you have to be there, there was not an option for virtual. It was [only] an option just to be there [in person]. That support I did not get, like saying, “Well today you can just go virtual and do whatever you can,” that kind of need. I need a mental health aid. I need to be flexible.( 42 year-old female, Nutritionist)*

Some employers facilitated flexible work hours, including consultation work, to accommodate diverse schedule needs.

*There are days I can’t do two hours a day. I’m down to an hour, or some days I can’t do anything. Luckily, I work for a justice organization, so they’ve been very supportive. They’re willing to take whatever I can give them at this point, and, as a consultant, that seems to have worked out. (53-year-old male* *,* *Nonprofit Director)*

Difficulty navigating state and federal support systems was a common theme among participants. Many expressed dissatisfaction with the processes for enrolling in Medicare, Medicaid, and unemployment benefits due to burdensome enrollment systems and the complexity of service options. Some participants were able to access service navigators who supported them through enrollment, without whom government services fell out of reach. This contributed to a sense of fatalism for some workers experiencing Long COVID.

*It was the worst. It was almost like it’s set up for people to fail at getting this help. … just [in] general, the government sites, like getting on unemployment. (40-year-old male, Actor/Singer)*

Overall, participants voiced a desire to remain in the workforce both for financial reasons and for their sense of identity. Most of the participants in this study were still employed despite their chronic symptoms but felt the stress of not meeting their previous level of productivity. Participants stated that their financial stability was precarious due to a lack of formalized protection. They called for employers to make necessary changes to accommodate employees in their return to the workforce.

*I have days where I could support from home. Heck, I might even be able to make it in once a week because I make it to doctor's appointments. I could come and check in. I really feel like my employers missed an opportunity to set an example… rather than behaving the way that they did in my condition... [They] had an opportunity to be the tip of the spear, I think, at reincorporating long haulers back into the workplace. It doesn't have to be an all-or-nothing thing…. A lot of what I did as a nurse in that office, I could do from home because it was a lot of talking to patients, explaining test results, helping triage their symptoms and things like that. (44-year-old female, Nurse)*

### Healthcare support

In addition to occupational and financial support, participants detailed their experiences accessing a range of healthcare resources throughout the chronic phase of illness. Access to provider visits (both in-person and virtual), specialty care, and receipt of pharmacotherapeutic treatments for symptom management were impacted by a range of barriers and facilitators described by the participants.

Providers lacked knowledge about Long COVID incidence, symptoms, trajectory, and possible treatments. Limited provider knowledge also undermined confidence in the medical system and frustrated participants who felt their symptoms were ignored and glossed over. In some cases, healthcare providers denied the existence of Long COVID, attributing symptoms instead to inadequate sleep, depression, or other factors. One participant who experienced severe persistent shortness of breath and chest pain recounted:

*The lung doctor they sent me to didn't think it was an issue. Just kinda said, "Ah, take some medicine for reflux," and that was it. I guess I felt like the way he acted that—almost kinda blew me off. He was very short. I don't know if he was stressed, or overstaffed, or whatever, but he was very short with me. Kind of dismissive of any concerns I might have that it was related to COVID. (50-year-old female, Profession Unknown)*

Providers’ lack of familiarity with Long COVID led to delays in referral or treatment. Some providers were aware of Long COVID as a phenomenon but seemed unsure of how to appropriately treat, refer, and support people experiencing Long COVID. Recognizing the need for specialty care to treat Long COVID symptoms by itself was not sufficient in bridging the gap to receiving care. Participants reported difficulty in scheduling specialty appointments despite referrals from their primary care providers. One participant pointed out the access barrier created by surges in COVID-19.

*My understanding is that the pulmonologists are just being swamped right now with people who have far worse symptoms than I do. I’m just like, yeah, how do I ever find out what the cause of this is? That’s just the frustration. I don’t know, maybe one day I can talk to my doctor. Maybe I can find a pulmonologist who has a free spot or something and talk to them*. *(61-year-old-female, Software Developer).*

Among participants whose providers acknowledged Long COVID, a lack of follow-up often prevented them from receiving needed care. One participant remarked on receiving several referrals to specialty care but received no additional instructions about the next steps. Many participants felt trapped by their Long COVID symptoms and without treatment options.

*“I told my primary doctor... ‘Hey, I noticed that COVID has bad brain fog,’ and they all say like, ‘We noticed that patients have that.’ I don't see any effort for referral or not even a medication needed to treat [it]. (42-year-old female, Nutritionist)”*

The data suggests a level of self-advocacy and determination were required to overcome the lack of care continuity; however, this ongoing burden was stressful, exhausting, and incredibly challenging to maintain.

*I have the feeling, given American healthcare, you have to be such a go-getter to figure this stuff out. If you just wait for your primary care doctor or even your pulmonologist, even these post-COVID clinics, you’d lose hope because you just don’t hear any—it’s crickets. It’s really crickets. To keep my own hope alive, I have to do this, or I’d be so down by now, I think. (53-year-old male* *,* *Nonprofit Director)*

While provider knowledge and behavior impacted participants’ ability to receive healthcare support, more systemic barriers played a distinct role as well. Insurance coverage, workers’ compensation for employees infected on the job, and required documentation created significant frustration for many participants experiencing Long COVID symptoms.

Participants had mixed feelings about the role of telemedicine in expanding access during Long COVID. While some acknowledged a loss of connection during the provider visit when conducted remotely, many relished the convenience of conducting appointments virtually:

*This is the best thing ever, because….I don't have to put on clothes. I don't have to get up off the couch. I can just lay here….That was really great. Then my next experience with telehealth was when I finally got an occupational health provider for my workers' comp claim. He started doing virtual visits with me probably about three, four months, probably four months into my illness. We've done virtual visits every two to four weeks for almost two years. (40-year-old female, Nurse)*

Additionally, participants reported that prescription services by mail, drive-thru windows, and curbside pickups helped them stay safe during the pandemic by decreasing in-person contact. While participants reported some success navigating the effects of COVID-19 and Long COVID, overwhelmingly participants experienced frustrations regarding lack of acknowledgement and adequate assistance from medical providers, not receiving appropriate or timely treatment, and a lack of clinical follow-up.

### Social and emotional support

Participants did not always differentiate between the social and emotional support that they needed and received in the acute phase of COVID versus the post-acute phase. In both phases, participants required support with physical tasks. During the acute phase, task-related supports such as meal and grocery drop-offs were essential due to the logistics of quarantine and were performed by friends, family, and co-workers. One participant discussed the support his family received in the first six weeks after he was diagnosed with COVID:

*When I had COVID, a lot of our friends and a lot of her friends really chipped in and helped the family out through meal trains and people dropping off things for the girls. It really helped. It took the burden off the family of trying to figure out how do we go to the store. I'm in quarantine; my wife's supposed to be in quarantine; how do we go to the store? How do we provide for our family? For many weeks or a month and a half, it really helped as a family dynamic. (43-year-old-male, Profession Unknown)*

The need for task-related support did not end with the acute phase of COVID. Delegation was necessary among the family, as family members experiencing Long COVID were unable to continue with the family-related tasks for which they had once been responsible.

*We are a lot more reliant on the older kids to help out because I can't do very much. I have good days and bad days. There are some days that I might be able to cook dinner or something like that. Then there's bad days where I have to be served my dinner at the couch because I just don't even have the energy to eat at the table. I* *can't even sit up and sit at the table and eat with my family. We have had to put a lot more on the kids because there's so many of them [kids]. (44-year-old female, Nurse & Graduate Student)*

Beyond this task-related support, family members and friends did not fully grasp the Long COVID experience. Participants often expressed a need for emotional support that was typically only found through others who shared the Long COVID experience, and they often connected through social media and support groups. Participants expressed a sense of comfort and hope in finding people who shared many of the same experiences, and Long COVID symptoms. Participants found online support groups especially helpful,

*[…] I was so scared. I was like—just with all the body changes, and all this weird stuff happening to your body, and the feelings, and so I joined that Facebook Long COVID group. I’ve got to tell you that made me feel so good, because there are … thousands of people who have the same exact symptoms that I had, and that was the first time I was like, “Oh, my God. I’m normal.” It gave me hope. (42-year-old female, Police Officer)*

Despite finding new social and emotional support for dealing with Long COVID, participants frequently discussed the barriers to engaging with their pre-pandemic social support structures. Participants often talked generally about social isolation that resulted from COVID protections during the pandemic, which were not unique to those experiencing Long COVID. However, people with Long COVID reported more complex barriers to social support related to their experience of the long-term consequences of COVID-19 and concerns around reinfection.

*I did have some people say. ‘This is all in your head. Just get out of bed, and get up and go exercise. Go move around, and you’ll get to feeling better.’ They didn’t realize that it was making me feel worse....Initially, it was a few friends of mine. I have a family member that said, ‘Hey, you just need to get up and get going. You’ll feel better if you go outside’. (56-year-old male, Director of Software Support Staff)*

### Overlapping themes

The thematic domains of support included overlapping features (see Fig. 1). These multidimensional facilitators and barriers of support are presented as additional themes.

#### Leveraging social networks for medical information

The social networks built through Long COVID support groups allowed participants to leverage existing relationships to keep current on COVID research. Specifically, several participants relied on support group members and family members in the medical field to help them find and critique the current COVID literature. A nurse who participated in the study discussed how her COVID support group worked to fill the Long COVID knowledge gap:

*We have a break off group, that's the data nerds working group where there's a lot of people that are either in the medical field or just plain out curious. Some of them are researchers, and a lot of people compile data there and so there's a lot of—we've done some of our own research. I think some of that's been published. Then also people will pull post articles and journals and things, and so it's just a one stop shop for you don't necessarily have to search it all out by yourself. I've read a ton of journals and articles there. (40-year-old female, Nurse)*

Another participant discussed how he had connected with other patients that he met at his Long COVID clinic to share research on possible treatments*,* allowing treatment conversations to take place between affected people instead of being limited to the clinical environment.*“I met some of his other patients, long-haulers, and we compared notes. We shared various case studies and reports that we were reading and seeing about long-haul treatment possibilities.” (53-year-old male* *,* *Nonprofit Director).*

Participants also expressed a desire to contribute to the growing Long COVID knowledge base by participating in research studies and contributing back to the growing community of people living with Long COVID,

*The other thing is, I think it’s important for those of us who have this to be able to reach out, or to search for research that they can be a part of […]. How are we going to figure all of this out, if people don’t volunteer to research? […] I think research is extremely important for long COVID. (55-year-old male, Director of Software Support Staff)*

#### Health insurance and healthcare costs

Participants discussed the dual role that employment status played, contributing to personal finances and healthcare access via employer-sponsored insurance benefits. Their employment was tied to health insurance, which is a gateway to healthcare in a privatized model. Even though they had sufficient insurance coverage, certain participants faced a lack of support in their Long COVID care due to access barriers arising from loopholes that affected individuals receiving disability or workers' compensation benefits. One healthcare worker who became infected with COVID-19 at work clearly articulated these barriers.

*I had to fight really hard to get in 'cause they are run ironically by the hospital that I'm affiliated with. At the time, they were only seeing patients that had their insurance, which of course I do but it was under my workers' comp claim, which is not the same insurance. I was like, "Okay, no, I'm gonna pitch a huge fit here. I got sick working for you guys so you are going to see me." They agreed to see me and said that they'll deal with the billing stuff later, which turned out to be a giant nightmare 'cause then of course, they turned around and billed me because workers' comp wouldn't pay for it, even though they had zero other options at the time. (41-year-old female, Nurse)*

Some employers did not support employees infected with COVID-19 at work; moreover, employee benefits such as workers’ compensation failed to provide the intended safety net. In this case, the employee was burdened with billing disputes related to treatment costs. Even participants who had insurance incurred financial costs of care such as co-pays and lab fees.

*I go to the doctor a lot, right? I have a lot of copays. I have a lot of tests that they run me through. I do have several thousand dollars of medical bills that I no longer had before. There is that side effect, or that burden. If that’s as bad as it gets economically, at least I still have a job. I still have insurance and stuff. (55-year-old male, Director of Software Support Staff)*

Due to the emergent nature of COVID-19 and its sequelae, many participants engaged in alternative therapies that required financial resources to access. They were driven outside of the healthcare system, which had no answers or relief for their Long COVID symptoms. Despite employment and insurance access, participants still lacked financial support for the costs of these treatments.

*I have to balance the amount of money I’m willing to pay ’cause a lotta this stuff is—you pay outta pocket. The supplements I’m on are expensive and maybe doing nothing –it's unclear. When you’re really sick, you’re willing to take risks that you wouldn’t normally take because your life already sucks, so maybe you’ll try something that normally you’d be like, “Well, am I gonna spend $200 bucks on that? No, I’m probably not,” and now, I might. (53-year-old male* *,* *Nonprofit Director)*

#### Social networks as a driver of financial stability

Participants who were unable to work due to acute or chronic illness experienced stress and instability around their finances. Those who were unable to work or whose income was reduced due to their disability relied on financial support from spouses, partners, and other members of their social networks. Most often, participants framed this as an economic and social disruption created by loss of household income. This change in household income contributed to shifts in responsibility, and financial worry.

*It did put more of a financial burden on myself, because at that point, I became the sole breadwinner, I guess. I don't know if that's the proper terminology, but the only income that was coming in was mine. That did add more stress, you know, because everything that we had done—the house that we were living in, our financial income—was based on a two-income household. Then all of a sudden, we went from a two-income household to a single-income household` (43-year-old male, Profession Unknown)*

Individuals bore the physical strain of Long COVID alone, but their spouses, partners, and family shared the financial burden of their loved one’s disability. One participant describes his financial situation: *“We can pay our rent, but we’re always a week late, so it’s like there’s an eviction notice on the door every time. It’s like we’re 12 h late, they definitely make it known.”* He then goes on to describe the toll that it played on his relationship. *“I had a lot of problems with my relationship with my fiancé… just feeling like we were both also so overwhelmed with things, because it’s like one thing after another.” (34-year-old male, Graduate Student).*

## Discussion

This study aimed to deepen our understanding of the Long COVID experience, with a specific emphasis on supports that facilitate improvement in Long COVID and possible reintegration into social and professional spheres. We identified three domains that clarify the support needs of people with Long COVID: (1) occupational and financial support; (2) social and emotional support; and (3) healthcare support. These domains do not exist independently, they are overlapping and interrelated. To adequately address the needs of the individuals included in this study, an integrated, holistic approach is necessary.

People with Long COVID and their families experienced significant financial hardship due to employment barriers and healthcare costs. This is echoed in the existing qualitative research, which identifies financial challenges as a key issue facing those experiencing Long COVID and a contributor to psychological distress [30]. Although participants wanted to return to work, the persistent symptoms of Long COVID frequently led to job loss or impaired their ability to perform required tasks. Employer accommodations were inadequate even for those who were able to maintain employment. Beyond financial distress and high healthcare costs, participants encountered diagnostic challenges, scarce specialty appointments, insurance loopholes, and medical skepticism, which drove their healthcare support needs. Although qualitative research among people experiencing Long COVID is limited within the U.S. healthcare system, these findings are shared across the U.K [30]. and Spain [31], identifying healthcare-related discrimination and stigma in European healthcare systems. Patients leveraged social networks to deal with these deficiencies in the healthcare and social service systems. Ultimately, improvement in the occupational and healthcare domains may alleviate the strain that chronic disability places on family and friends in the social sphere.

Addressing these systemic issues requires major shifts in the way medicine is practiced and social services are regulated [32]. Fortunately, numerous policy recommendations already exist aimed at addressing the support needs identified in this analysis. In April 2022, President Biden signed a presidential memorandum to create and strengthen support services for Long COVID treatment and for workers with Long COVID [33]. As part of the federal government’s acknowledgement of Long COVID as a disability under the Americans with Disabilities Act (ADA), the Department of Labor has issued guidance on supporting employees with Long COVID. The operationalization of this memo continues to develop along with our understanding of Long COVID. Advocacy groups have proposed policy recommendations and have advanced the TREAT Long COVID Act [34], CARE for Long COVID Act [35, 36], and the Long COVID Recovery Now Act [37] to secure funding and enact policy changes for Long COVID research and social support systems. The Department of Health and Human Services produced the Health + Long COVID Human Centered Design Report [38], which had multiple suggestions that require policy support, as did a report from the Wellesley Institute [32]. Advocacy groups also advanced suggestions for research priorities [39], calls for Long COVID Centers of Excellence [40], and integration of research and advocacy in the infection-associated chronic illness space, including ME/CFS and dysautonomia [39]. Based on this study’s findings and review of relevant policy documents, we suggest a 5-pronged approach for future policy development.

### Improve public awareness

Long COVID information must be presented as an inherent, vital part of COVID-19 messaging. Many participants in this study were frustrated by time-limited empathy from friends and colleagues who did not understand the chronic and ongoing nature of Long COVID. Participants experienced pressure from acquaintances and loved ones to “push through” their symptoms to their detriment. A recent analysis shows that there is a growing public interest in Long COVID information [41]. Advocates suggest declaring a Long COVID public health emergency with regular public information updates from the White House that would help to increase the public’s awareness of the prevalence, characteristics, and impact of Long COVID [34–36, 42]. Long COVID messaging should be targeted toward marginalized groups who may have less access to health information [32, 39]. Messaging should be culturally and linguistically appropriate for diverse groups to ensure the uptake of public health campaigns. Public information updates would also provide easy access to reliable, scientifically-sound information. Awareness campaigns should include symptom management and make recommendations based on available resources [35, 36, 38].

### Improve clinical care quality and access

A recent study found that of people accessing Long COVID clinics, 40% traveled over one hour to access care, and more than 50% waited between one and three months for an appointment [43]. Our participants affirm the lived experience of these findings, expressing frustration about a lack of available, quality services. People with Long COVID need expanded access to virtual or geographically convenient locations, rapid appointments, and knowledgeable providers. The President’s emergency plan for AIDS relief or PEPFAR could be a model for a permanent Long COVID response that includes agency-based guidelines for clinicians, ongoing clinical education, healthcare facility training to increase access, and increased Long COVID education in medical and nursing schools [44]. A similar model would also help to streamline the clinical definition of Long COVID and mandate reimbursement for care standards [45]. More nuanced ICD-10 codes related to PEM or post-exertional symptom exacerbation (PESE), the pathognomonic symptom of myalgic encephalomyelitis/chronic fatigue syndrome (ME/CFS) and common in people with Long COVID have been proposed to track treatment and improve service quality within marginalized communities and assure access to quality treatment [46].

### School and workplace accommodations

Loss of employment among study participants and the need for additional workplace accommodations are demonstrated in the existing quantitative literature. Davis et al. (2021) showed that 22% of people experiencing Long COVID were not able to work, and another 45% needed reduced work schedules. [16] Because Long COVID appears to be more prevalent in people of working age compared to older adults, this impact on productivity has the potential for significant economic loss [47].

Our participants expressed specific needs for flexible work hours and options for remote work; difficulty accessing unemployment benefits and other social support services; and a desire for physical support to help with fatigue management. These needs are consistent with the “opportunities” identified in the Department of Health and Human Services Health + Long COVID report. Broader policy suggestions for addressing needs include creating government guidance regarding appropriate accommodations at school and at work [34–36, 38], and involving occupational therapists and vocational rehabilitation specialists in determinations around reasonable accommodations [38]. Suggestions specific to individual workplaces include a change of role, reduced work hours, and the opportunity for remote work [32, 38, 48]. Additionally, workplaces can shift their focus to meeting goals or targets rather than hours spent in the office or online. This can help workplaces accommodate individuals’ needs for activity management strategies like symptom-contingent pacing.

### Strengthen economic benefits and social services

Our study participants were older and more financially stable than the average American, and they may not fully represent minoritized or marginalized populations. Given the documented racial, ethnic, and income disparities in COVID-19 [49], it is important to consider the needs of these underrepresented populations during Long COVID policy creation and implementation. We anticipate that many people with Long COVID may benefit from expanded access to public benefit programs, such as the Supplemental Nutrition Assistance Program (SNAP), Temporary Assistance for Needy Families (TANF), Medicaid, Supplemental Security Income (SSI), and Social Security Disability Insurance (SSDI), especially if they continue to be unable to work. This is consistent with needs identified and policies suggested by multiple advocacy organizations [48]. Our participants discussed a need for assistance navigating existing programs suggesting a need for funding for Long COVID-literate, trauma-informed caseworkers to guide applicants through these programs [48].

Policy recommendations also included government organizations ensuring people with Long COVID had the ability to go on and off disability without reapplication [38]. Further, government programs could shoulder some of the economic burden of long-term disability on employers through programs like tax credits or loan forgiveness [38].

### Improve research and data collection on Long COVID

Currently, there are many publications describing the epidemiology and symptomology of Long COVID, but there is a dearth of published research on Long COVID diagnostics and treatment. Participants in our study looked to alternative and unstudied treatments for Long COVID in their need for symptom relief. Increased federal funding for Long COVID research will help facilitate interventional research; and fast-tracking research for pharmaceutical interventions that have shown promise in infection-associated chronic illnesses, such as ME/CFS, postural orthostatic tachycardia syndrome (POTS), or mast cell activation syndrome (MCAS), will bring the possibility of relief to millions living with Long COVID [39]. Additionally, long-term, methodologically-sound Long COVID cohort studies are needed to better understand Long COVID onset after initial infection, reinfection, infection based on variant, and vaccination, as well as the course, symptomology, and effective management of Long COVID [46].

Although we discussed recommendations in several disparate policy areas, an integrated approach is necessary for effective and efficient COVID-19 policy implementation. This integration is not limited to policy professionals and public officials, but must also include employers, healthcare providers, as well as those living with and impacted by Long COVID.

## Limitations

There was a lack of racial/ethnic and socioeconomic diversity in this study. We are cautious in our interpretation of these findings because of the history of erasure of people marginalized by race, ethnicity, and economics in research and medicine. Further studies that focus on and center the experiences of people of color are warranted, as are studies that feature participants with different socioeconomic backgrounds. Additionally, the focus on cardiovascular symptoms during recruitment for the parent study may have led to a sample that skewed older, with more male participants, than the current Long COVID demographics suggest [16].

Additionally, because this was a qualitative sub-analysis nested within a parent study with separate aims and scientific underpinnings, the interview guide was not structured specifically to understand experiences of support. This analysis was inductive and exploratory. Thus, the results may not include the full breadth of perspectives on this topic. Future research should seek to comprehensively explore the support and access needs of people with Long COVID.

### Supplementary Information

**Additional file 1.** Synthesis of Long Covid Policy Recommendations.

## Data Availability

Data cannot be shared publicly because it contains protected health information, and due to IRB confidentiality and data sharing restrictions. Data are available from the Johns Hopkins Medicine Institutional Data Access / Ethics Committee (contact via Phone: 410–502-2092 or E-Mail: jhmeirb@jhmi.edu) for researchers who meet the criteria for access to confidential data.

## Abbreviations

ADA: The Americans with Disabilities Act
COVID-19: Severe Acute Respiratory Syndrome Coronavirus 2, or SARS-CoV-2
GED: General Education Development
Long COVID: Post-Acute Sequelae of COVID-19 Infection
MCAS: Mast Cell Activation Syndrome
ME/CFS: Myalgic Encephalomyelitis/Chronic Fatigue Syndrome
PASC: Post-Acute Sequelae of COVID-19 Infection
PEM: Post-Exertional Malaise
PESE: Post-exertional symptom exacerbation
POTS: Postural Orthostatic Tachycardia Syndrome
SNAP: Supplemental Nutrition Assistance Program
SSI: Supplemental Security Income
SSDI: Social Security Disability Insurance
TANF: Temporary Assistance for Needy Families
USD: United States Dollar ($)
