# Supplementary material for: “It was almost like it’s set up for people to fail” A qualitative analysis of experiences and unmet supportive needs of people with Long COVID
Source: BMC Public Health. 2023 Oct 31;23:2131. doi: 10.1186/s12889-023-17033-4 (PMC10617090; doi:10.1186/s12889-023-17033-4)
Supplement: Supplementary file 1 — Additional file 1. Synthesis of Long Covid Policy Recommendations. [file 12889_2023_17033_MOESM1_ESM.pdf]

SYNTHESIS OF LONG COVID POLICY RECOMMENDATIONS

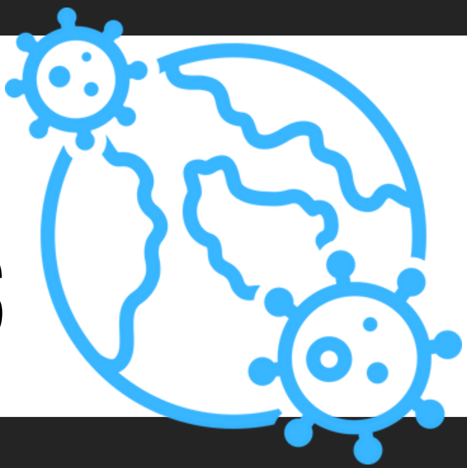

01 INCREASE PUBLIC AWARENESS

Advocates are concerned about the devaluation and omission of long-term COVID-19 sequelae in government-funded materials. Long COVID information must be presented as an inherent, vital aspect of COVID-19 messaging.

02 IMPROVE CARE QUALITY AND ACCESS

Many patients report low-quality and ineffective care from Long COVID clinics. Providers need smart, evolving, up-to-date clinical education; patient feedback should be used as a metric for care improvement; and care access should be improved, particularly in marginalized and minoritized populations.

03 SUPPORT SCHOOL AND WORK ACCOMMODATIONS

Long COVID often presents as an episodic disability, making it challenging to predict if and when return to work is appropriate. Flexibility from both employee and employer is key. Given the high prevalence of Long COVID in people of working age, a robust workplace safety net is necessary, including sick leave, disability benefits, and accommodations.

04 STRENGTHEN SOCIAL BENEFITS AND SERVICES

Current social service systems are already strained. The current system must be reinvigorated by increased staffing. The current system must be reinvigorated through increased staffing, guidance around public assistance, and simplification of existing applications and procedures.

05 IMPROVE RESEARCH AND DATA COLLECTION

Research showing promise for other infection-associated chronic illnesses should be fast-tracked and epidemiologic studies that increase our understanding of Long COVID should be prioritized. Patient-led research and patient inclusion in study design are essential.

01 INCREASE PUBLIC AWARENESS

- Create public information campaigns centering Long COVID [1–4]
- Declare Long COVID a public health emergency with regular public information updates from the White House [4]
- Use communication science research to develop the campaign and inform approach [3]
- Focus on messaging to marginalized communities [2,3]
- Provide information on prevalence, characteristics, and impact of Long COVID [1,5,6] and illnesses often comorbid with Long COVID [1,4]
- Provide information on how to manage symptoms, including information and treatments, and how to obtain care and services [1,7]
- Develop materials in conjunction with experts in the advocacy, clinical, and research spaces, including those with expertise in associated conditions [1,2,7]

02 IMPROVE CARE QUALITY AND ACCESS

Improving Care Quality

- Establish a permanent entity to aid in prevention, research, and treatment of Long COVID, modeled after PEPFAR, the President's Emergency Plan for AIDS Relief [4]
- Integrate experts and research from ME/CFS (myalgic encephalomyelitis/chronic fatigue syndrome), dysautonomia, and other infection-associated chronic illnesses which are being diagnosed in those with Long COVID [3,5,7–9]
- Create a government agency-led consensus-based clinical guidelines and care pathways and algorithms for clinicians [7]
- Create a system of smart, evolving, and up-to-date education in clinical care [1,2,4–7] and disability documentation[8], including useful advice on CPT codes for providers.[7] All materials should be grounded in historical successes and failures in addressing infection-associated chronic illness,[1,7] including emphasis on biological etiology [4] and an understanding of Long COVID's chronicity.[8]
- Offer training incentives for healthcare facilities and medical providers to learn about Long COVID and other infection-associated chronic diseases[7]
- Support Long COVID clinical education in medical schools and offer additional reimbursement for providers to see 'complex' cases[7]
- Employ a user rating system to guide quality improvement (QI) research[7] and require annual reports from those who receive grant funding for Long COVID that incorporate patient evaluations [5]
- Incorporate new ICD codes for Long COVID [9] and for symptom post-exertional malaise (PEM), the pathognomonic symptom of ME/CFS, shared by the majority of patients with Long COVID [10]

Improving Care Access

- Conduct a needs assessment for care, treatment and support, evaluating the role of structural barriers in access to care, particularly in marginalized communities [3]
- Prioritize interventions in system navigation for medically underserved populations and those disproportionately affected by COVID-19 [2,5], including ensuring Long COVID patients can continue to receive telehealth medical appointments after the PHE is over [7]
- Establish Long COVID clinical centers and Centers of Excellence [5,7,11] and provide funding to train community health centers in Long COVID care, with integrations between medical providers, researchers and case managers [7]
- Train Long COVID care managers to help schedule and prepare patients for appointments [7]
- Ensure that Medicare and Medicaid will support treatments and interventions with success in Long COVID and associated chronic illness [7]
- Ensure treatment cannot be denied based on insurance coverage, date or method of diagnosis, or prior hospitalization [5] and offer training incentives for health insurers to learn about Long COVID and other infection-associated chronic diseases [7]

03 SUPPORT SCHOOL & WORKPLACE ACCOMMODATIONS

- Create materials outlining Long COVID as a disability and clarifying Long COVID patients' rights at work and at school [1,5]
- Create government guidance regarding appropriate accommodations at school and at work [1,5,7] involving occupational therapists and vocational rehabilitation specialists in determinations around reasonable accommodations.[7] Workplaces can provide accommodations, a change of role, reduce work hours, make accommodations for disease prevention (such as ventilation), allow the opportunity for remote work [2,7,8] and provide disability-friendly workstations in the office.[4] Additionally, workplaces can shift their focus to meeting goals or targets rather than hours spent in the office or online, to accommodate patients' need to use pacing, and allow workers to go on and off disability without reapplication.[7]
- Direct patients to job search and training programs in cases where the individual remains too ill to return to their previous position [7]
- Create compensation schemes for employing people with disabilities, in the vein of the Wounded Warriors and Returning Heroes tax credits, compensating those who contracted COVID-19 and ultimately, Long COVID, at their job [7]
- Create loan forgiveness programs and other accommodations for students with Long COVID [7]
- Create and support a process for determining that employers and schools are reasonably accommodating people with Long COVID, especially given that marginalized and low-wage workers are disproportionately exposed and are less likely to have the protections above[2]

04 STRENGTHEN SOCIOECONOMIC BENEFITS & SOCIAL SERVICES

- Create guidance around epidemics and pandemics for public benefit programs[8]
- Perform targeted outreach to multiply marginalized groups regarding access to public benefit programs[8]
- Congress should increase funding for Long COVID-literate, trauma-informed[8] caseworkers to guide applicants through public benefit programs[7,8] and the medical system[7]
- Make public benefit program applications more accessible by:
  - creating a single application for multiple programs;[8]
  - offering multiple ways to apply: in person, online, and by phone;[8]
  - improving public benefits websites including creating save-points, progress bars[8], shortening the application[7], and using other tools for applicants with limited energy; and
  - enmeshing Long COVID clinics with public assistance and disability support services to create a 'one-stop shop'[7]
- Make Social Security credits available for those with Long COVID and their caregivers[7]
- Accept documentation verifying a medical condition from a wider range of medical providers and from other professionals, such as social workers or homeless service providers[8]
- Resolve SSA staff shortages to process applications in a timely manner.[7,8] In addition, SSI SSDI applications, approvals and denials should be tracked for Long COVID and associated conditions[3,4], and CMS should collect data on items and services furnished to those enrolled in Medicaid or CHIP for Long COVID.[1] SSDI applications tagged with Long COVID may also be fast-tracked.[7]
- Change the CPS Disability Supplement questions in order to encompass all disabled people, including those with episodic disabilities like Long COVID[12]
- Make funding available to advocacy groups working in the Long COVID space from private donors and foundations. Increase charitable giving write-offs to 100% of one's adjusted gross income to incentivize charitable giving from American taxpayers[7]
- Congress should:
  - Remove program work requirements and time limits for TANF and SNAP[8]
  - Remove asset caps for TANF, SNAP, Medicaid, SSI, and SSDI[8]
  - Remove the requirement of Continuing Disability Reviews (CDRs) every seven years for people enrolled in SSI in instances where individuals have severe medical cases with no opportunities for significant improvement.[8]
- State agencies should revise disability evaluations to "take into account lack of workplace accommodations and access to/ability to take public transportation, in determining if an individual is unable to feasibly substantially work to meet program requirements"[8]
- The Administration for Community Living should award grants to establish and/or expand medical-legal partnerships to provide individuals with Long COVID and associated conditions effective aid. Funding should be available not just to states but also to nonprofit advocacy organizations, Indian Tribe or Tribal organizations, and healthcare providers impacted by COVID and Long COVID [1]

05 IMPROVE RESEARCH & DATA COLLECTION FOR LONG COVID AND ASSOCIATED CONDITIONS

- NIH should establish an Office of Complex Chronic Conditions Research (OCCCR) [3,4], authorized to establish research priorities for Long COVID and all complex chronic conditions, including ME/CFS, fibromyalgia, MCAS, Ehlers-Danlos syndrome, and dysautonomia.[3,4] The office should be responsible for developing research priorities[3] and charter an Office of Complex Chronic Condition Research Advisory Council as a Federal Advisory Committee.[3]
- NIH should develop a strategic plan for Long COVID and other chronic complex conditions, integrating experts and research from other infection-associated chronic illnesses[3]
- Increase research funding for Long COVID[4,7], including "high risk, high reward" research[7] on interventions supported in other chronic infection-associated conditions [3], excluding potential harmful treatment paths such as talk therapy and exercise therapy[4]
- Prioritize multi-year epidemiology studies to track Long COVID[3,7], and make the results publicly available.[3] These should include disparities of Long COVID diagnosis disaggregated by demographics and the possibility of onset after initial infection, reinfection, infection based on variant, and vaccination.[3]
- Invest funds in the areas of highest scientific priority based on the strategic plan, identifying opportunities and addressing gaps[3]
- Special focus should be given to studies in minoritized groups [3], vaccination-associated cases [3,4], and children [3], and to studies with special focus on post-exertional malaise[3]
- Federally-funded studies should meet recommended targets for recruitment for race, age, ethnicity, sex, gender (including transgender and nonbinary people).[3] RECOVER should improve their recruitment to produce a more diverse, representative group [3,7]
- Require meaningful patient engagement at every step of the research process for federally-funded studies [1,3], rather than focusing on later stages such as recruitment, patient engagement, and translational processes [3], and require fair compensation for this engagement.[1,3] Ongoing studies, such as RECOVER and INSPIRE should improve their patient engagement process [3]
- Accelerate research prioritized by Long COVID and post-viral illness experts [3]
- Accelerate one-year trials into Long COVID that have shown promise in associated conditions and work with the pharmaceutical industry to identify and address barriers[3]
- Ensure all federally-funded Long COVID studies meet best practices for study design:
    - Use WHO definition, which includes suspected cases [3]
    - Include comprehensive symptom tracking, including post-exertional malaise, cognitive symptoms, and menstrual symptoms [3]
        - PEM should use standard definitions and instruments recommended by NINDS Common Data Elements
- Care should be taken with EHR data studies, which are invariably biased towards:
    - Patients with more severe presentation, with positive COVID tests, and with healthcare access [3]
    - Patients with specific, potentially incorrect diagnostic labels (such as POTS misdiagnosed as anxiety) [3]
    - More patients with overt organ dysfunction (respiratory/cardiac issues) and fewer with syndromic, multi-system conditions [3]

- The US Census Bureau and Bureau of Labor Statistics should collect data to assess the impact of Long COVID on the labor market and how best to accommodate workers, including adding new questions to disability surveys targeting Long COVID and associated conditions [3,8]
- New ICD Codes for post-exertional malaise (PEM), the pathognonmic symptom of ME/CFS shared by many with Long COVID [10] and an ICD Code for Long COVID should be created [9]

*This document is a synthesis of existing policy recommendations and does not necessarily represent the opinions of all manuscript authors

References

1. Kaine T. CARE for Long COVID Act [Internet]. S. 801 Mar 15, 2023. Available from: https://www.congress.gov/bill/118th-congress/senate-bill/801?q=%7B%22search%22%3A%5B%22CARE+Long+COVID%22%5D%7D&s=1&r=2

2. Sanford S, Wadehra R. Long COVID: Literature Scan to Inform Policy Response in the GTA [Internet]. Wellesley Institute; 2022 Nov. Available from: https://www.wellesleyinstitute.com/wp-content/uploads/2022/11/Long-COVID-Policy-Response-2022-Final.pdf

3. Patient-led Research Collaborative, Strategies for High Impact, The Myalgic Encephalomyelitis Action Network, Marked by COVID. Towards a Patient-Driven National Research Action Plan: Recommendations for the National Research Action Plan on Long COVID [Internet]. Available from: https://patientresearchcovid19.com/storage/2022/06/Towards-a-Patient-Driven-National-Research-Action-Plan.pdf

4. Long COVID Action Project. Help End the Long COVID Crisis (U.S. Letter) [Internet]. Available from: https://actionnetwork.org/letters/help-end-the-long-covid-crisis

5. Pressley A. TREAT Long COVID Act [Internet]. H.R. 7482 Apr 7, 2022. Available from: https://www.congress.gov/bill/117th-congress/house-bill/7482?s=1&r=95

6. GAO Science, Technology Assessment, and Analytics. Science & Tech Spotlight: Long COVID [Internet]. U.S. Government Accountability Office. 2022. Available from: https://www.gao.gov/assets/gao-22-105666.pdf

7. Department of Health and Human Services, Office of the Assistant Secretary for Health. Health+ Long Covid Human Centered Design Report [Internet]. 200 Independence Avenue SW, Washington, D.C. 20201; 2022. Available from: https://www.hhs.gov/sites/default/files/healthplus-long-covid-report.pdf

8. Burnside A, Lower-Basch E, Dolby T, Gilkesson P, McCorkell L. Advancing Disability Equity and Access in TANF and SNAP for People with Long COVID [Internet]. The Center for Law and Social Policy; 2022 Oct. Available from: https://www.clasp.org/wp-content/uploads/2022/10/2022.10.12_Advancing-Disability-Equity-and-Access-in-TANF-and-SNAP-for-People-with-Long-COVID.pdf

9. Patient-led Research Collaborative. Feedback on ICD-10-CM proposal for U09.9 [Internet]. 2021. Available from: https://patientresearchcovid19.com/storage/2022/10/PLRC-ICD-10-CM-Long-COVID-Comment.pdf

10. Patient-led Research Collaborative. Proposal to add ICD-10-CM code for post-exertional malaise/post-exertional symptom exacerbation [Internet]. [cited 2023 Apr 9]. Available from: https://docs.google.com/document/d/1dotGidT7XY8gX9AnwR0rsaiMZH3-mEDd/edit

11. The Myalgic Encephalomyelitis Action Network. Call-to-Action for California: Centers of Excellence [Internet]. 2022 May. Available from: https://www.meaction.net/2022/05/02/call-to-action-for-california/

12. Patient-Led Research Collaborative, The Myalgic Encephalomyelitis Action Network. Response to Request for Information on Current Population Survey Disability Supplement 2024 [Internet]. 2022. Available from: https://www.meaction.net/wp-content/uploads/2022/08/PLRC-and-MEAction-CPS-Disability-Supplement-Public-Comment.pdf
